# Current Trends in Nanotheranostics: A Concise Review on Bioimaging and Smart Wearable Technology

**DOI:** 10.7150/ntno.82886

**Published:** 2023-03-11

**Authors:** Amit Kumar Sharma

**Affiliations:** Department of Chemical Engineering, National Tsing Hua University, Hsinchu, Taiwan

## Abstract

The area of interventional nanotheranostics combines the use of interventional procedures with nanotechnology for the detection and treatment of physiological disorders. Using catheters or endoscopes, for example, interventional techniques make use of minimally invasive approaches to diagnose and treat medical disorders. It is feasible to increase the precision of these approaches and potency by integrating nanotechnology. To visualize and target various parts of the body, such as tumors or obstructed blood veins, one can utilize nanoscale probes or therapeutic delivery systems. Interventional nanotheranostics offers targeted, minimally invasive therapies that can reduce side effects and enhance patient outcomes, and it has the potential to alter the way that many medical illnesses are handled. Clinical enrollment and implementation of such laboratory scale theranostics approach in medical practice is promising for the patients where the user can benefit by tracking its physiological state. This review aims to introduce the most recent advancements in the field of clinical imaging and diagnostic techniques as well as newly developed on-body wearable devices to deliver therapeutics and monitor its due alleviation in the biological milieu.

## Introduction

The scope of various therapeutic approaches towards physiological ailments has widened in recent times 1-3. Conventional therapeutic models such as targeted drug delivery systems are now experiencing a paradigm shift by the technological advancements in disease prognosis and diagnosis 4-6. The elevated standards of medical technology have brought revolution through smartphones and electronic health that is becoming inclusive to the conventional medical practice 7,8. Together with smartphones, the convenience of sharing patient health records with the healthcare workers through electronic health platforms is changing the patient treatment experience through remote treatment facilities 9. Theranostics is such an approach that is focused on therapeutic interventions together with diagnostic tools to facilitate continuous monitoring of the physiological condition in a patient.

## Bioimaging

Bioimaging has proved to be an accurate and reliable diagnostic method owing to the visual aid in observing damaged tissue or organ 10. Technological advancement over the years in *in vivo* imaging system have overcome the challenges of autofluorescence, low resolution, and closed-circuit camera by improving the penetration depth of the incident laser, that has resulted in non-invasive imaging modalities 11. In this journey, functional nanomaterials have emerged as an alternative to the conventional organic contrast agents which are known for their detrimental effects on the biological tissues when over exposure 12. Several different kinds of nanomaterials with fluorescence properties in visible to near-infrared I and II emissions are developed and employed as contrast agents for imaging 13,14. In this section, we will go through the conventional bioimaging tools that facilitated the visual observation of some of the common physiological conditions followed by the advanced tools with high resolution imaging capabilities. This review will also discuss the applicability of such imaging tools and new contrast agents that have been proposed to use in theranostics and continuous monitoring of the physiological state.

Historically, the application of imaging techniques, that were originally developed for space applications, began in 1960s where researchers explored the possibilities of optical imaging, X-ray imaging and computed tomography (CT), magnetic resonance imaging (MRI) for inspecting food and agricultural products. Further advancements by assembling optical cameras, appropriate illumination and developing computer software began the era of bioimaging that opened a new research area. Currently acceptable clinical imaging techniques such as Positron emission tomography (PET), CT-scan, MRI, ultrasound imaging, or photoacoustic imaging have their own advantages and limitations.

### Positron emission tomography (PET)

**Figure 1:**
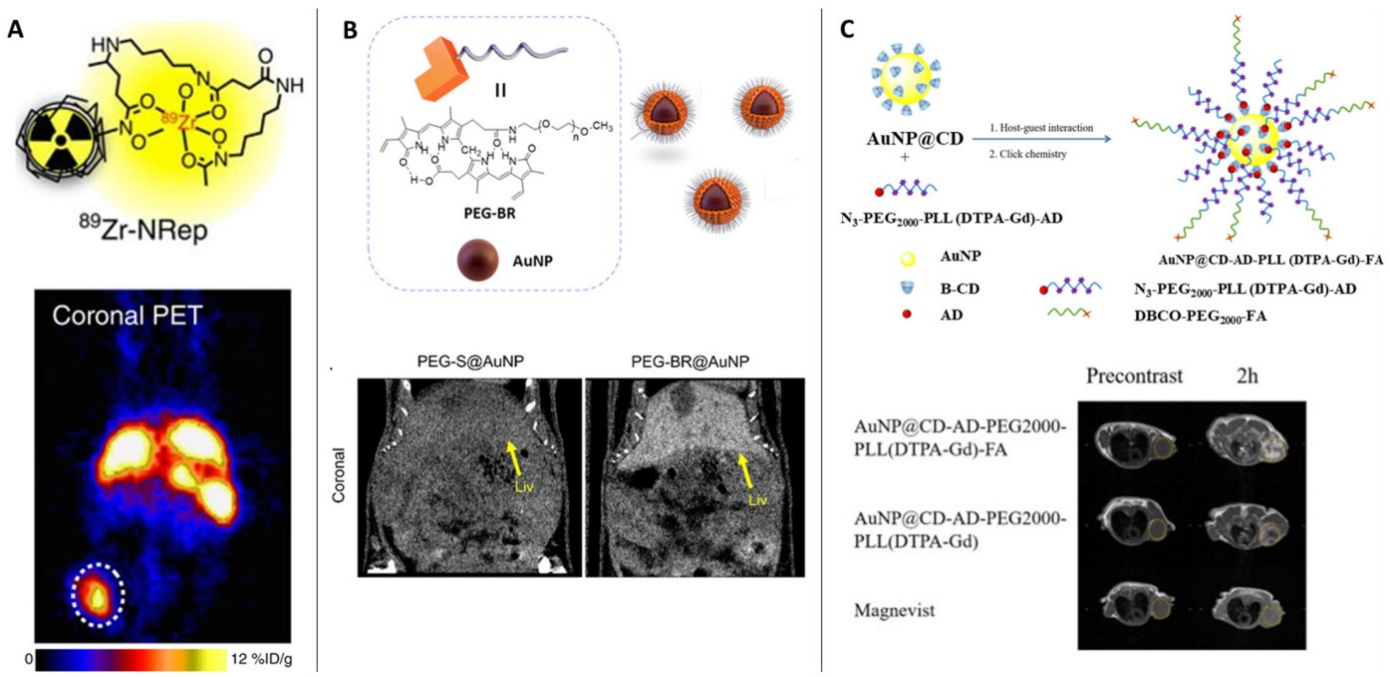
** (Column A)** The liposomal nanoreporter ^89^Zr-NRep modified with ^89^Zr-chelating desferrioxamine (DFO) used as PET contrast agent; Representative images of 4T1 tumour-bearing mice with high 89Zr-NRep uptake 3. Copyright 2016, Nature. **(Column B)** Schematic illustration of the fabrication of polyethylene glycol (PEG)-bilirubin (BR) protected gold nanopartilces (AuNPs) as glutathione-sensitive CT contrast agent; Representative images of the abdomens of healthy mice administered PEG-BR@AuNPs compared to thiolated PEG-S@AuNPs shows bright contrast with PEG-BR@AuNPs in the liver 24. Copyright 2021, American Chemical Society. **(Column C)** Schematic representation of the multifunctional AuNP-based MRI contrast agent protected by cyclodextrin, 1-adamantanecarbonyl chloride, polyethylene glycol, lysine and diethylenetriaminepentacetate acid bound gadolinium conjugated with folic acid (AuNP@CD-AD-PEG2000-PLL(DTPA-Gd)- FA); Comparison of magnetic resonance images of tumor-bearing mice injected with different contrast agents (yellow dot: tumor region) 30. Copyright 2022, Elsevier.

PET is one the most sensitive imaging tools that can reveal functional images at the molecular level with high resolution. Owing to its sensitivity, PET has been employed for various ailments such as cancer, neurodegenerative diseases, coronary artery and assessing myocardial variability. The operation of PET relies on the detection of the gamma rays originating from the annihilation of the positrons emitted from the radioactive molecule introduced in the patient. Among the most popular radioactive molecules, ^18^F-fluorodeoxyglucose (FDG) is prepared by selectively replacing the oxygen atom by ^18^F, which is further metabolized by the cells revealing the metabolic processes following the Warburg effect. Similarly, ^11^C-L-methionine amino acid is used to identify cancer in the biological environment 15. The radioisotopes used in PET often have long half-life and decay time, thus damaging vital organs of the patient. Recently various forms of ^64^Cu labelled nanoparticles have been reported as an efficient alternative demonstrating shorter half-life and biocompatibility than the conventional probes 16. While the artifact signals generated due to the free ^64^Cu^2+^ is still a challenge, its doping with other metals prevents aggregation of the probe while extending circulation times and easy excretion. A recent report on the use of ^89^Zr conjugated Doxil as a nanotheranostic agent was used to assist in imaging the tumor recession and monitor the efficacy of the drugs in PET 17,18 (Figure 1A). The use of radiolabeled probes as PET agents requires functionalization of the drug molecules to the probes to enable dual role 19.

### CT-scan

The clinical challenges associated with PET scan such as exposure to radioactive materials and biocompatibility are slightly resolved in computed tomography (CT) scans which are derived from X-ray imaging. Compared to the use of radioactive probes in PET, CT scans functions by collecting and analyzing the cross-sectional X-ray signals that are retrieved from the contrast agents injected in the patient several hours before the test. To obtain high-resolution images, the scan time is longer which necessitates the longer retention and circulation time of the contrast agents. The renal clearance of the contrast agents and prolonged circulation are achieved by controlled size of the contrast agents (larger than the glomerular filtration barrier) and partial diffusion in the blood. The success of the CT scan is justified by its ability to differentiate between soft tissues, water, and bones. Apart from barium sulfate, several different contrast agents based on iodine 20, bismuth 21; use in picture), gold nanoparticles 22, liposomes 23 have been employed as CT scan contrast agents. Yoo et al showed the fabrication of glutathione-sensitive gold nanoparticles designed by protecting the surface with polyethylene glycol (PEG)-bilirubin (BR) to obtain serum stable contrast agent (Figure 1B). The GSH sensitivity of the contrast agent was exploited to differentiate between healthy liver from a diseased liver through the CT scan 24.

### MRI

While PET and CT scans require ionizing radiation for biological imaging, causing serious side effects on human health, magnetic resonance imaging has overcome some inherent problems associated with observing the skeletal and neuronal tissues 25,26. The use of radiolabeled molecules that respond to the incoming X-ray radiation to generate images of the target organ is often observed to stay longer in the biological system and takes time in clearance from the body. MRI has proved relatively safe owing to the biocompatible contrast agents that is another advantage to high-resolution imaging 27,28. Conventional contrast agents based on gadolinium are observed to induce acute side effects such as nausea to severe side effects 29, its biocompatibility and efficacy has been improved by its functionalization on gold nanoparticles. A host-guest interaction-based gadolinium assembly on beta-cyclodextrin-modified gold nanoparticles are employed to localize, accumulate, and image tumor region with excellent biocompatibility was reported by Zhang et al 30 (Figure 1C). Also, in a pilot clinical trial, sentinel lymph node melanoma was imaged through the superparamagnetic iron oxide to overcome the short-half-life of the radiolabeled probe ^99m^Technetium, which requires single photon emission computed tomography (SPECT-CT) and a tedious standard operating procedure 31. Despite the inherent challenge to apply MRI as theranostic agents, a significant contribution aiding to its applicability is reported based on the response of the contrast agents against pH, temperature, enzyme, redox reactions, ultrasound, and near infrared light 32.

While these imaging modalities are currently used in clinical practice, the demand for efficient and biofriendly contrast agents with ease of handling is growing. Due to the rise in electronic health and precision therapy, theranostics tools capable of monitoring physiological conditions are gaining popularity. Several innovative researches have brought diagnosis and therapy together with patient-controlled delivery of therapeutic agents, thereby facilitating personalized medication. The following section shows the advancements in the direction of theranostics and patient compliance.

### Ultrasound and photoacoustic imaging

**Figure 2:**
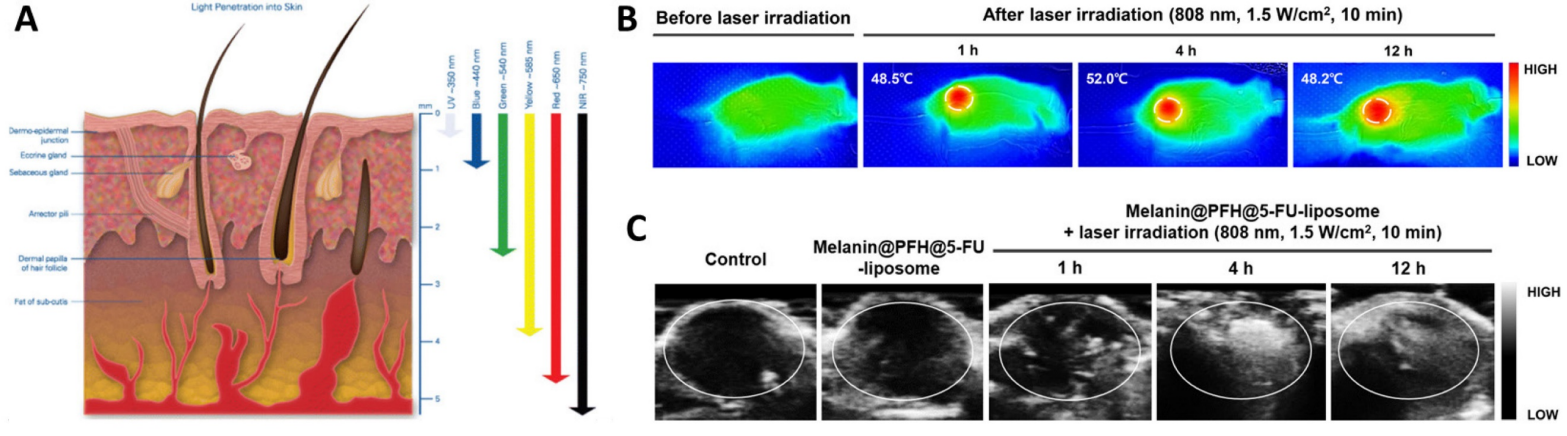
** (A)** Illustration showing the depth to which human skin is penetrated by light. While blue and ultraviolet light scarcely enter tissue, red light extinguishes at 4-5 mm beneath the skin's surface 33 Copyright 2017, Springer. **(B)** IR thermal images showing Melanin, Perfluorohexane (PFH) and 5-fluorouracil (5-FU) loaded liposomes (melanin@PFH@5-FU-liposomes) generating heat upon NIR 808 nm light. **(C)** Ultrasound images of tumor tissues in CT26-bearing mice before and after laser irradiation at 1 h, 4 h, and 12 h after intravenous injection of melanin@PFH@5-FU-liposomes 40. Copyright 2022, Springer Nature.

As highlighted above, the clinical application of probe-based imaging tools poses a health risk due to their radioactivity and biocompatibility. On the contrary, light and sound waves are relatively much safer alternatives to gather information from biological system. The spectrum of light encompassing UV, Vis, NIR, IR, are known to penetrate under the skin and reach deeper tissues and organs (Figure 2A) 33. Sound waves on the other hand due to their longer wavelength, can penetrate deep tissues easily without any significant optical obstruction due to skin or microscopic cellular environment 34. Supplemented with appropriate contrast agents, ultrasound imaging generates high-contrast images comparable to other imaging tools 35. Owing to its interaction and absorption by the biological tissues, ultrasound can also cause temperature rise, resulting in activation of the immune response or cellular death due to ablation 36,37. This phenomenon is widely explored in designing sonodynamic therapy for cancer and non-invasive glucose regulation for treating diabetes 38,39. In a recent study, the peripheral-focused ultrasound method was demonstrated to stimulate the hepatoportal nerve plexus to alter glucose concentrations in the blood. This method was tested on several animal models for chronic stimulation that can reverse the onset of hyperglycemia by targeting the liver-brain pathway 39. Ultrasound irradiation causes the production of cavitation-based microbubbles in the biological environment which is explored in ultrasound-assisted bond cleavage for releasing drugs from liposomes 40 and inorganic nanoparticles 41.

Various sonosensitizers producing reactive oxygen species in an aqueous environment have been employed to treat cancer 42. Inorganic nanoparticles such as TiO_2_ and Zr-MOFs are excellent sonosensitizers; their efficiency has been improved by introducing other transition metal groups like Fe_3_O_4_ to form Janus nanostructure 43 or creating oxygen-deficient surfaces 44. TiO_2_ is popularly known as a sonosensitizer; however, it suffers from low transduction efficiency due to the rapid recombination of the electron-hole pairs. Owing to its biocompatibility, improving the sonodynamic transduction efficiency to produce ROS will be promising for cancer therapy and theranostics applications. The coating by Fe_3_O_2_ on TiO_2_ improves the transduction efficiency by narrowing the bandgap of TiO_2_, thereby reducing the recombination rate. Similarly, ZrO_2_-deficient nanoparticles showed higher efficiency in producing ROS under the US due to the more significant separation of the electron-hole pairs. The biocompatibility of ZrO_2-x_ nanoparticles was improved by capping with amine-polyethylene glycol with high absorption in the NIR-II window. It is seen that the NIR-II irradiation also triggers ROS generation in the biological tissue, which is an intriguing approach to employing NIR-II-based therapeutic activation and imaging. Despite the ease of operability of ultrasound imaging in observing SDT activity 40, the technique primarily suffers from low resolution and interference from surrounding tissues (Figure 2B and 2C). Technological advancements such as bio adhesive ultrasound are intriguing approach for on-body continuous organ monitoring 45. The device is made of a rigid ultrasound probe coupled in-between a soft hydrogel elastomer hybrid, that can transmit high acoustic waves and prevent its detachment from the skin. The bio adhesive ultrasound probe could adhere on the skin and stays without deformation for over 48 hours.

Molecular imaging is another upgrade in medical imaging providing information about the biochemical processes in the biological milieu. The resulting images overcome the limitations of poor spatial resolution and radiolabeling in other imaging tools. Based on its sensitivity towards nanocontrast agents, photoacoustic imaging has been employed in molecular imaging for several applications. J-aggregates of organic dyes are known to exhibit emission in longer wavelengths that further translate to photoacoustic imaging contrast agents. Indocyanine green (ICG) are also shown as a potential contrast agent due to its stable J-aggregates which are encapsulated in a liposome for high sensitivity and estimating saturated oxygen in blood vessels 48. Other contrast agents such as gold 49,50, silver 51, copper 52, zinc selenide 53, iron 54, cyanine dyes 55 are employed in imaging brain related pathological conditions 56, tumor volumes 57, CAR-T cells 58.

**Figure 3:**
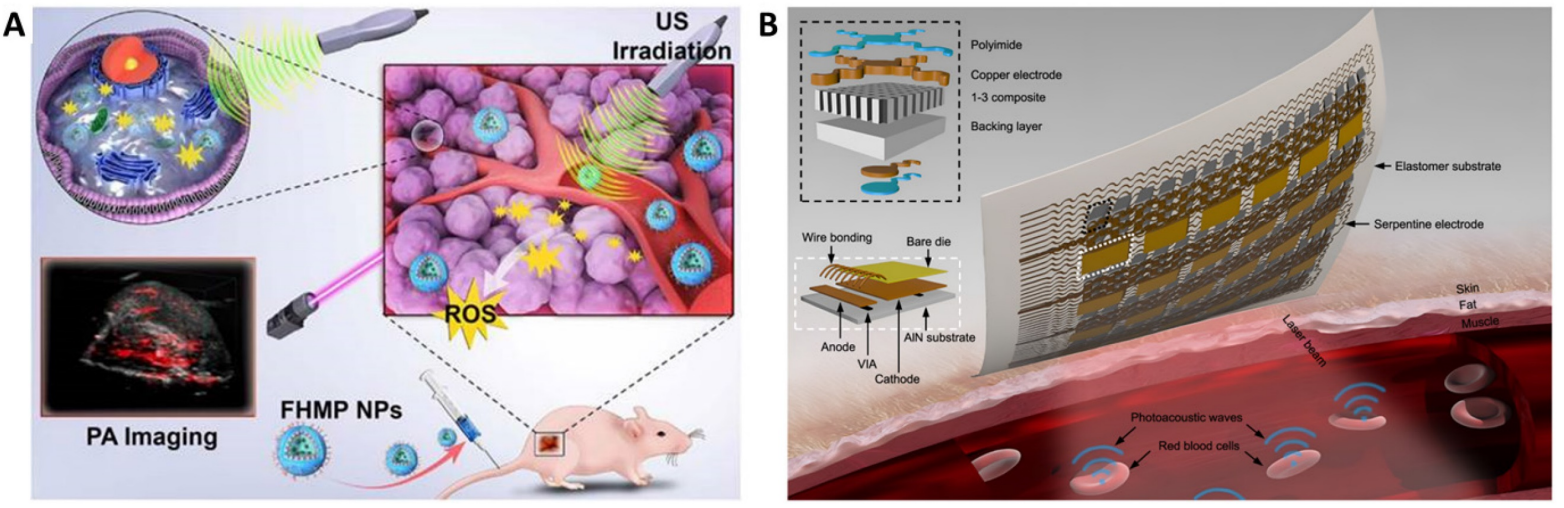
** (A)** Schematic illustration of the multifunctional nanoplatform for efficiently photoacoustic imaging-guided sonodynamic therapy to tumor cells/tissue 47. Copyright 2018, IvySpring. **(B)** Schematics of soft-photoacoustic patch for detection of hemoglobin and measuring temperature. The hemoglobin molecules in red blood cells go through a thermoelastic expansion after absorbing optical energy, which then transmits acoustic waves into the surrounding media. The transducer array will gather the photoacoustic waves and then transmit them to a backend system for data processing 60. Copyright 2022, Nature.

The success of photoacoustic imaging for diagnosis still relies largely on the efficiency contrast agents. However, the need for high intensity laser light to facilitate skin penetration may lead to low resolution due to optical attenuation. Other factors such as targeted tissue localization and ultrasound obstruction through gas cavities or lung tissues affect the resolution of the image 59. Addressing its sensitivity towards optical absorption and ultrasound generation, Gao et al developed a wearable continuous monitoring epidermal patch. The vertical-cavity surface-emitting laser diodes (VCSEL) arranged in an array on the epidermal patch can generate laser pulses to penetrate the skin and a piezoelectric transducer array for detecting the resulting ultrasound waves. The hemoglobin in the blood expands due to the localized heating under incident laser thus causing an acoustic wave which is detected by the piezoelectric transducer. Such an epidermal patch can generate high spatial resolution image of single molecules (Figure 3B) 60.

### Light-based theranostics

Light based imaging modalities based on fluorescent nanomaterials are another approach to visualize biological processes and structures in living cells and tissues 61,62. These materials have unique optical and physical properties that make them ideal for bioimaging applications, such as high sensitivity, selectivity, and the ability to be functionalized with specific biomolecules. Examples of nanomaterials used in fluorescence bioimaging include quantum dots 63, gold nanoparticles 64-66, and fluorescent dyes 67. Quantum dots, which are also semiconductor nanocrystals, have particularly high fluorescence efficiency and broad absorption spectra, making them useful for multiplexing and deep tissue imaging 68,69. Gold nanoparticles have strong light scattering properties and can be used to enhance the signal of fluorescent probes 70,71, while fluorescent dyes can be used to label specific biomolecules or structures 72. The use of nanomaterials in fluorescence bioimaging is expected to have a significant impact on the early diagnosis and treatment of diseases, as well as on our understanding of biological processes at the molecular level.

Fluorescent nanomaterials have been at the forefront of the bioimaging-based tracking of biological tissues. Various functional groups have been reported to facilitate the functionalization of nanomaterials, acting as a probe for a visual aid of the targeted tissue. Targeting adipose tissues is a significant challenge due to their widespread availability as a protective layer surrounding vital organs and metabolic activity. Brown adipose tissue, among the other types, viz. Beige and White adipose tissue, actively metabolize fatty acids when subjected to low temperatures. Owing to the inherent fatty acid composition, the adipose tissue is non-responsive to alternate detection methods such as electrical signals, thus, leaving an optical or ultrasound-based approach 73. Taking advantage of the fatty acid metabolism and its inherent affinity towards adipose tissue, Paulus et al. reported the applicability of a BODIPY functionalized fatty acid tracer for PET and fluorescence imaging 74. The in-situ esterification of the surface fatty acid to triglyceride, followed by its entrapment into chylomicron-like particles, assists its absorption by the intestines and traverses through the lymphatic system to the brown adipose tissues. When subjected to varying temperatures to instigate fatty acid metabolism in the brown adipose tissue, the BODIPY dye is activated, resulting in sub-cellular imaging of the adipose tissue. A decade of research on the progression of cancer is now correlated to the surrounding adipose tissue, primarily brown adipose tissue 75,76. Hypoxia instigating therapeutics and radiotherapy have made cancer cells strive for fatty acid metabolism pathway for survival through molecular signaling. The adipose tissue surrounding tumor undergoes fatty acid metabolism and produces energy through TCA cycle, that causes the patient with lean body and loss in weight due to excess fatty acid metabolism. Taking advantage of the surrounding adipose tissue, Wen et al proposed a formulation composed of rumenic acid, doxorubicin prodrug, fatty acid binding protein conjugated at a radical sensitive chemical bond, which is loaded on adipose cells and locally delivered at tumor site 77. Owing the fatty acid binding protein and rumenic acid, the formulation can be stored in the adipose cells, and H_2_O_2_ catalyzed separation of the prodrug causes tumor cell death, resulting in highly efficient therapy. Conjugating fluorescent probe on similar designs could facilitate a theranostics solution to monitor the treatment progress of the tumor.

Using optogenetics, researchers have developed methods to release therapeutic genes using visible light from smartphone as a convenient source to activate the release process, with the goal of making treatment more patient friendly. A recent study by Mansouri et al 78 has shown that the green light from smartwatch is able to remotely control transgene activation and demonstrate the release of human glucagon-like peptide-1 from engineered human cells to treat diabetes. Such an approach is highly promising as the current available smartwatches can project green and red light (corresponding to 540 nm and 690 nm), which is biofriendly and could be used to design various theranostics approaches.

Current fluorescence-based bioimaging modalities employing nanomaterials as contrast agents suffer from certain limitations such as altered physicochemical properties post-interaction with the biological entities *in situ*, photo instability of the probes, long duration of the imaging, photon scattering and tissue absorption at the visible or near infrared region of the spectrum with poor spatiotemporal resolution 79,80. To overcome these challenges, nanomaterials capable of absorbing in the NIR-II region of the spectrum, with high emission intensities are developed using lanthanides, carbon nanotubes, quantum dots, and organic-inorganic complex. The tunable biocompatibility of the next generation NIR-II probes enables its applicability as a stimuli-responsive therapeutic delivery vehicle as well as an efficient probe to the biochemical reactions 81-83. Owing to its facile electron energy transfer in the NIR-II region, multiplex layers of lanthanide elements are explored in the field of bioimaging producing high resolution images of blood vessels in animal models 84,85.

## Nanotechnology and theranostics

Wearable devices are being developed for the therapeutic treatment of various organs and systems in the body. For example, wearable devices are being used to deliver insulin to patients with diabetes 86, to provide pain management for individuals with chronic pain conditions 87, and to deliver targeted chemotherapy to cancer patients 88,89. Wearable devices are also being developed for the treatment of cardiovascular diseases, such as by delivering medications or providing electrical stimulation to the heart 90-92. In the field of neurology, wearable devices are being used to deliver brain stimulation to treat conditions such as Parkinson's disease and depression 93,94. These devices have the potential to improve patient compliance and outcomes, as they allow for continuous or intermittent therapy while the patient goes about their daily activities. Wearable therapeutics are an emerging field with great potential to revolutionize the way that many medical conditions are treated. In this section we will see the current trends in the integration of smartphone technology and electronic health in theranostics.

### Wound healing

**Figure 4:**
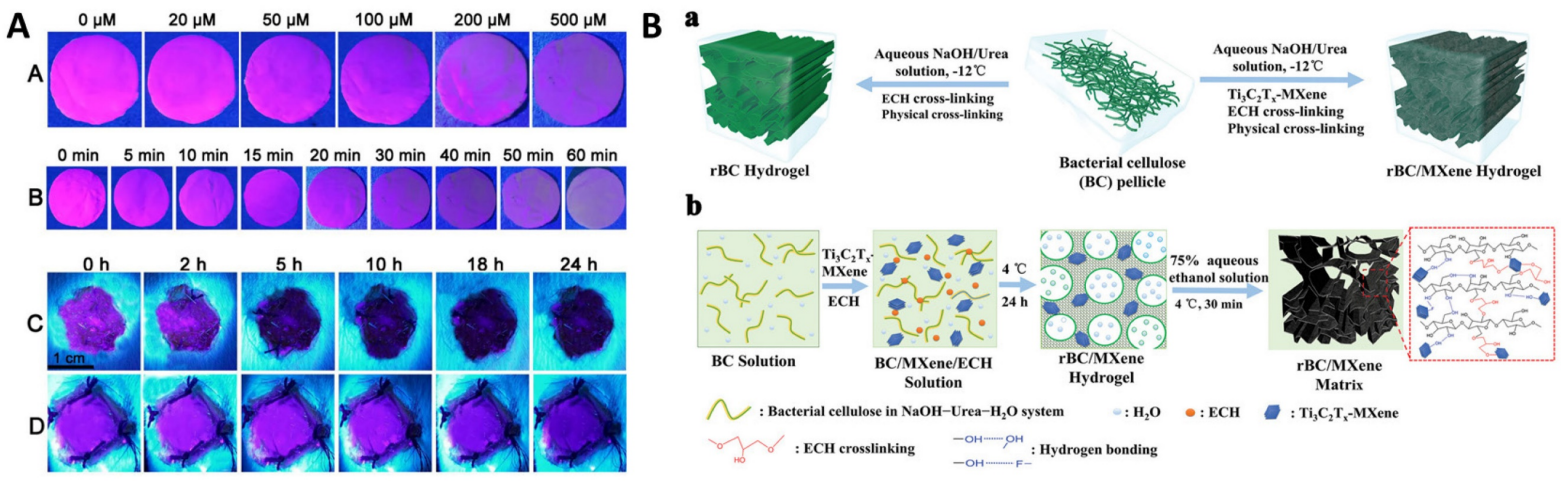
** (Panel A)** Fluorescent color change in Polyacrylonitrile (PAN)-europium (Eu) coordination polymer mats at excitation wavelength 397 nm: (A) with different concentration of H_2_O_2_, (B) with different incubation time of H_2_O_2_ (200 μM); (C) on the wound after bacterial non-infection and (D) on the wound after bacterial infection 109. Copyright 2020, Elsevier. **(Panel B)** (a) Schematics representation of the fabrication of regenerated Bacterial Cellulose (rBC)-based hydrogels incorporating Ti_3_C_2_T_x_ MXene through chemical and physical crosslinking; (b) Diagram of the mechanism of synthesizing rBC/MXene composite hydrogels 119. Copyright 2020, Wiley.

The biomolecular reactions occurring at the wound site during the wound healing process are categorized into four stages, viz. hemostasis, inflammation, proliferation, and remodeling. While hemostasis lasts a few minutes post injury, inflammation and proliferation are the determinants of the wound status and its healing capabilities which lasts from 5 to 20 days. In the early two stages of wound healing, blood clotting is followed by platelets degranulation at wound site promotes the release of a series of mediators from basophils and mast cells causing vasodilation, which further instigates the secretions of fibrinogen and other chemoattractant resulting in swelling of the wound. During proliferation, tissue granulation and angiogenesis begin to contract the wound area with the secretion of several growth factors while in the remodeling stage, the endothelial cells along with macrophages remodel the wound area by replacing damaged cells through apoptosis 95. On the contrary to normal wound, chronic wound is among serious terminal diseases due to impaired healing process, which is characterized by complications in wound debridement and biofilm formation due to wound dressings. Current wound management is primarily focused on regular debridement and reapplication of absorbable or non-absorbable dressing based on the site of the wound. Conventional wound dressings made of fabric loaded with antibiotic and stimuli responsive biomolecules 96 are now being replaced by biological alternatives such as fish scales, organic hydrogel 97, DNA hydrogels 98, organic and inorganic nanomaterial-hydrogel composite dressings 99,100, oxygenated wound site 101 through oxygen releasing microspheres loaded in polymer matrix 102 and other approach 103-105. Among the plethora of biomarkers secreted in the wound exudate 106, dynamic concentrations of H_2_O_2_ are associated with the healing stages 107,108. The concentration of H_2_O_2_ is significantly high in the hemostasis and inflammation stage while gradually reducing through the proliferation and remodeling stages. Owing to its dynamic localized concentrations, H_2_O_2_ has attracted the development of smart composite dressings that can exhibit functional properties in the form of fluorescence or electrical signals. Wu et al developed europium coordinated polymers loaded into polyacrylonitrile nanofiber mats which can absorb the wound exudate and exhibit varying fluorescence intensity after interaction with localized H_2_O_2_ concentration (Figure 4A) 109. Moisture retention, absorption of the wound exudate and antibiotic are necessary factors to promote healing of chronic wounds and prevent biofouling. Wound debridement and periodic change of dressing are deemed necessary to aid in rapid healing. Promoting cellular migration of endothelial cells and macrophage to the wound site assists in the proliferation and remodeling stages. It is seen that electrical stimulation over the epidermis could attract these cells to the wound site and promote rapid healing 110-112. To facilitate electrical stimulation through wound dressing, conducting polymers (such as PEDOT:PSS 113, PAni 114), triboelectric nanogenerators 115,116 are incorporated into hydrogels. The composite dressings are integrated with electrical signal measuring tools and bridged to smartphone for continuous monitoring of the healing progress 117,118. MXenes are recent exploration in this direction of electroactive wound dressing, embedded in bacterial cellulose to promote cellular proliferation and migration at the wound site 119,120. In a typical fabrication, as shown in Figure 4B (a), the bacterial cellulose (BC) pellicles were resolved using NaOH and epichlorohydrin (ECH) cross linker to obtain regenerated bacterial cellulose (rBC). The physical and chemical cross linking of Ti_3_C_2_T_x_ MXene into the BC solution in the presence of ECH resulted in rBC/MXene matrix conducting hydrogel (Figure 4B (b)). In another approach, Kalidasan et al have demonstrated a wirelessly controlled sutures made of PEDOT:PSS conducting polymer which operates at 1-2 GHz to monitor deep surgical wounds 121.

### Vagus nerve

The vagus nerve is a long, complex nerve that extends from the brainstem to the abdomen and controls a wide range of functions, including heart rate, digestion, and the immune system 122. Monitoring the vagus nerve can provide important information about the body's physiological state and can be used to diagnose and treat a variety of medical conditions. For example, vagus nerve stimulation (VNS) has been used to treat epilepsy 123, depression^124^, and other conditions 125 by delivering electrical impulses to the nerve. In addition, the vagus nerve is involved in the body's response to inflammation and monitoring the vagus nerve can provide insights into the immune system and the development of inflammatory conditions 126,127. The vagus nerve is also a key component of the parasympathetic nervous system, which helps to regulate rest and relaxation, and monitoring the vagus nerve can provide insights into stress and other psychological factors 122. Overall, the vagus nerve is a critical part of the body's overall functioning and monitoring it can provide important information for the diagnosis and treatment of a wide range of medical conditions.

Due to its association with Parkinson's disease, epilepsy, clinical depression, and gastroparesis, biomedical engineers are interested in tapping the potential of vagus nerve to address these challenges. The current clinical implant is placed at the lower neck to stimulate and monitor the vagus nerve in a critical surgical procedure. Alternatively, transcutaneous vagus nerve stimulation is at the forefront in clinical trials as an effective non-invasive approach 128-130. Another alternative to battery powered metallic implants is based on triboelectric nanogenerators. Graphene-loaded polyacrylamide hydrogel triboelectric nanogenerator (HENG) 131 were shown to generate alternating electric current of 1.6 mA after exposed to 0.3 W/cm^2^ focused ultrasound through vibration induced compression in the electric double layer of the hydrogel. Due to its self-powered battery-free and biocompatible property, such technology holds great promises to remote controlled vagus nerve stimulation and monitoring. The innervation of the vagus nerve in the visceral adipose tissue and the gut region makes it an attractive therapeutic approach to alleviate the psychological and physiological condition of the patient 130,132,133.

### Obesity

While it is well known that adipose cells tend to exist in the body for long periods, slow rate of triglyceride removal against its gradual buildup over the years is major cause of obesity 134. In recent years, obesity has turned to be a major public health problem that is associated with a range of negative health outcomes, including diabetes, heart disease, and stroke. Adipose tissue, or fat, is a key factor in the development of obesity, and imaging techniques can be used to visualize and measure adipose tissue in the body. There are a number of imaging techniques that can be used to assess adipose tissue, including computed tomography (CT), magnetic resonance imaging (MRI), and ultrasound 135. CT and MRI can provide detailed images of fat tissue and allow for the measurement of fat volume and distribution. Ultrasound can also be used to measure fat thickness and to evaluate the structure of fat tissue. In addition, newer techniques such as positron emission tomography (PET) and single photon emission computed tomography (SPECT) can be used to measure metabolism and blood flow in adipose tissue. These imaging techniques can provide important information about the distribution and function of fat tissue and can be used to evaluate the effectiveness of weight loss interventions 133.

Despite various approaches to the treatment of obesity, there is still room to develop an effective theranostics approach demonstrating effective drug delivery and simultaneous detection adipocyte shrinkage 136.

### Depression and mental health

In recent years, chronic stress and subsequent depression is a growing concern of modern society affecting a large population across the globe. The severity is many folds when its identification is highly subjective and where attempts to correlate the levels of chemical signaling molecules in the blood with the clinical diagnosis for depression are on the rise 137. Cortisol, a steroid hormone is an active biomarker for physiological as well as psychological stress 138. High levels of cortisol suggest chronic stress with signs of anxiety and depression along with altered blood pressure, blood glucose and a plethora of physiological ailments. Clinical therapeutics in the form of suppressants pose a threat of addiction and severe damage to vital organs. Thus, it is imperative to keep track of stress hormones, or the therapeutics administered to regulate the levels of cortisol. Contrast to point-of-care biosensors, wearable devices and micro patches are gaining immense popularity due to its real-time information and overcoming the artifacts in fluorescence or optical sensors methods. Parlak et al have shown the fabrication of wearable electrochemical device for identifying cortisol from sweat 139. Conductive polymers, such as PEDOT:PSS, come handy for the fabrication of on-body wearable patches for sweat-based biomarker detection. For the fabrication of the on-body cortisol sensor, organic electrochemical transistor composed of PEDOT:PSS was prepared with an electrolyte solution for gating. The OECT would transduce the cortisol ions entering into the sensor into electrical signal through molecularly imprinted polymers as receptors to the neutral charge cortisol. Tang et al demonstrated the applicability of a Prussian blue oxidation-based on-body stretchable sensor for cortisol 140. The sensor was prepared using polypyrrole deposition on molecularly imprinted polymer electrodes in presence of cortisol along with Prussian blue dye, followed by elution of cortisol from the membrane. The elution of cortisol from the membrane creates a cavity resulting in current flowing through the Prussian blue dye. When cortisol from sweat is absorbed back to the cavity, the oxidation current flowing through the dye in polypyrrole membrane is reduced, thus correlated to the cortisol levels in the sweat. The scope of applicability of micro patches or sensors to monitor biomarkers from sweat is widening and offers great promises to integrate with smartphones or other electronic health devices.

### Electroceuticals

Electroceutical are categorized as therapeutics targeting neural circuits of the internal organs 141. Treatment examples include vagus nerve stimulation 142, spinal cord stimulation 143, cochlear 143 and retinal 144 stimulation through biocompatible implants. This approach is extended in targeting chronic lung disorders 145, hypertension 146, diabetes and gastrointestinal diseases 147. Implants based electrical stimulation have been proved effective to certain diseases such as cardiovascular pacemakers, the next generation of electroceuticals are more targeted towards self-powered and biodegradable implants. As cited previously, hydrogel nanogenerators are developed for vagus nerve stimulation 148. The innervation of pain receptors in the adipose tissue underlying the subcutaneous opens new avenues for non-invasive detection and stimulation of these electrical signals that will provide information about the user's physiological condition 149. Among the other methods to read the electrical signals such as voltage, resistance, current or tissue penetrating light through a wearable device, impedance is based devices are relatively easy to calibrate for measuring subcutaneous or deep tissue electrical signals 150. Nanomaterials and injectable composite hydrogels for localized electrical stimulation coupled with bioimpedance measurements could prove an effective approach for therapeutic delivery and continuous monitoring of the disease condition.

### On-body wearable monitoring systems

Wearable devices have the potential to revolutionize the way that physiological challenges such as cancer are monitored and treated. These devices can continuously monitor a patient's vital signs and other health parameters, such as temperature, oxygen saturation, and heart rate. Wearable devices can also be used to deliver targeted therapy to cancer patients, such as by releasing chemotherapy drugs directly to the site of cancer 151,152. This can help to minimize side effects and improve patient outcomes. In addition, wearable devices can be used to monitor the effectiveness of cancer treatments, such as by measuring the size of tumors over time. Overall, wearable devices have the potential to improve cancer care by providing real-time monitoring and treatment and by enabling personalized medicine approaches. A pioneering effort towards continuous monitoring of tumor volume was presented by Abramson et al 153. A thin elastomeric film was developed as a conformal, wearable strain sensor that, when placed on the skin of a mouse-bearing subcutaneous tumor, would deform from the original strain due to the corresponding volume of the tumor. The working principle of the strain sensor relies on the change in electrical resistance of the drop-casted 50 nm gold layer on top of the elastomer. As the tumor volume changes in dimensions, the electrical resistance in the gold layer is changed. Owing to the percolation network formed in the elastomer, the resistance in the sensor changes exponentially, corresponding to the tumor volume. This method is up-and-coming in the direction of tracking the biological activity in the skin and potentially translates to other epidermal challenges.

Several new approaches in the direction of nano theranostics in lung cancer are highlighted in this review 154. Cuffless measurement of blood pressure is a new challenge in wearable devices that would enable the integration of the method into smartphones and smartwatches for a more compliant and quick way to keep track of cardiovascular health. Bioimpedance is a viable approach capable of identifying electrical signals from deep tissues, which is a significant advancement to other methods based on optical, acoustic or pressure sensors. Popular smartwatches available to the general population can detect heart rate but are inefficient to detect blood pressure due to limited penetration depth and signal retention from deep tissue. Kireev et al have addressed this problem by designing a cuffless monitoring system for arterial blood pressure using graphene tattoos through bioimpedance 155.

## Outlook and Conclusion

Clinical imaging techniques are powerful tools that are used to visualize and diagnose a wide range of medical conditions. However, these techniques also have certain limitations that need to be considered. For example, many clinical imaging techniques involve the use of ionizing radiation, which can be harmful to the patient if used excessively. Some imaging techniques, such as computed tomography (CT) and nuclear medicine, are particularly high in radiation dose, and care must be taken to ensure that the benefits of the examination outweigh the risks 156. Other limitations of clinical imaging techniques include their cost, which may not be accessible to all patients, and their availability, as some specialized equipment may only be found at large medical centers. In addition, some imaging techniques, such as magnetic resonance imaging (MRI), are contraindicated for patients with certain medical implants, such as pacemakers. Finally, the interpretation of imaging studies requires specialized training and expertise, and incorrect interpretation can lead to misdiagnosis and inappropriate treatment.

For cancer theranostics and obesity, it is now shown that the Brown adipose tissue is closely related to cancer progression and overcoming hypoxia-based treatment hindrance in the tissue. BODIPY-based fluorescent nanoprobe have been shown to target the adipose tissue, primarily the brown adipose tissue, that opens new avenues towards cancer theranostics. Nanomaterials with properties of targeting the adipose tissue and exhibiting fluorescent response upon excitation under NIR-II laser could be a new direction to tackle cancer. Photoacoustic imaging is another promising tool to observe and narrow down the field of vision to blood cells and single cells. Coupled with ultrasound imaging or electrical impedance, such methods can be useful for monitoring the treatment progress. Interventional nanotheranostics encompassing both therapeutics and diagnosis/monitoring of the treatment progress is a promising and enticing approach that would aim at patient compliant and patient friendly treatment.
